# Immunogenic cell death (ICD) genes predict immunotherapy response and therapeutic targets in acute myeloid leukemia (AML)

**DOI:** 10.3389/fgene.2024.1419819

**Published:** 2024-08-14

**Authors:** Shuang Wen, Xuefeng Lv, Xiaohan Ma, Shu Deng, Jinming Xie, Enwu Yuan

**Affiliations:** ^1^ Reproductive Center, The First Affiliated Hospital of Zhengzhou University, Zhengzhou, Henan, China; ^2^ Department of Laboratory Medicine, The Third Affiliated Hospital of Zhengzhou University, Zhengzhou, Henan, China; ^3^ Zhengzhou Key Laboratory for In Vitro Diagnosis of Hypertensive Disorders of Pregnancy, Zhengzhou, Henan, China; ^4^ Deyang Prison, Deyang, Sichuan, China

**Keywords:** AML, immunogenic cell death, pseudogene, prognosis, survival, immunotherapy, chemotherapy, prognostic model

## Abstract

**Introduction:**

Numerous studies have demonstrated acute myeloid leukemia (AML) is one of the malignancies with high mortality worldwide. Immunogenic cell death (ICD) is a form of cell death that is specialised in that it triggers the body’s immune response, particularly the adaptive immune response. Recent evidence has confirmed that pseudogenes are implicated in multiple human tumorigenesis and progression although lacking the function of coding protein. However, the roles of ICD-associated genes in AML remain largely unascertained.

**Methods:**

TCGA-AML and GSE71014 cohorts were picked out and we combined them into a merged dataset by removing the batch effect using the sva package in the R project. A consensus clustering analysis of the ICD genes in AML was performed to define subgroups. Based on the expression of 15 prognostic-related pseudogenes, we developed a prognostic model and categorized AML samples into low and high-risk groups.

**Results:**

AML was differentiated into two subgroups (C1 and C2 clusters). Most ICD-related genes were significantly up-regulated in the C2 cluster. The single sample gene set enrichment analysis (ssGSEA) revealed that the immune cell infiltration and immune checkpoint gene expression of the C2 cluster was strongly high, suggesting that the C2 population responded well to immune checkpoint blockade (ICB) therapy and had better survival. The C1 group was sensitive to chemotherapy, including Cytarabine, Midostaurin, and Doxorubicin. On the other hand, 15 ICD-related pseudogenes were identified to be associated with AML prognosis. The receiver operator curve (ROC) analysis and nomogram manifested that our prognostic model had high accuracy in predicting survival. However, the high-risk group was sensitive to ICB therapy and chemotherapy such as Methotrexate, Cytarabine, and Axitinib while the low-risk group benefited from 5-Fluorouracil, Talazoparib, and Navitoclax therapy.

**Discussion:**

In summary, we defined two subgroups relying on 33 ICD-related genes and this classification exerted a decisive role in assessing immunotherapy and chemotherapy benefit. Significantly, a prognostic signature identified by critical ICD-related pseudogene was created. The pseudogene prognostic signature had a powerful performance in predicting prognosis and therapeutic efficacy, including immunotherapy and chemotherapy to AML. Our research points out novel implications of ICD in cancer prognosis and treatment approach choice.

## 1 Introduction

Acute myeloid leukemia (AML) is defined as a heterogeneous malignancy where myeloid cell differentiation is inhibited and uncontrolled proliferation of leukemic blasts (Liu, 2021; Newell and Cook, 2021). In adult diagnoses, AML is the most prevailing acute leukemia and led to cancer-related deaths with a median age of 68 years at the diagnosis stage (Koenig et al., 2020). Accumulating evidence has manifested that there is a wide range of genomic mutations and alteration (Döhner et al., 2017; Rio-Machin et al., 2020). AML can be separated into multiple biologic subtypes relying on cytogenetic abnormalities and genetic mutations. Notably, these classifications play a determining role in survival time and final clinical outcomes in AML. The risk stratification guidelines of AML have been broadly employed to assess prognosis outcomes (Pogosova-Agadjanyan et al., 2020). As a result, dissecting genetic abnormalities is beneficial to develop new biomarkers and therapeutic targets for anti-AML treatment.

Immunogenic cell death (ICD) is defined as a cell death pattern that is sufficient to mobilize innate and adaptive immune responses within the immunocompetent context, which finally promotes the formation of sustaining immunological memory (Galluzzi et al., 2020; Birmpilis et al., 2022). The antigenicity and adjuvanticity carried by fading cancer cells determine the immunogenicity that has influenced the anti-tumor response (Bloy et al., 2017). The antigenicity of tumor-associated antigens (TAA) has limited immune activity when lacking supplementary adjuvants that contribute to the activation and function of antigen-presenting cells (APC). Dying cancer cells release some signal molecules that serve as the damage-associated molecular patterns (DAMPs) in the cellular demise, which induces tumor‐specific immune responses (Hayashi et al., 2020). The innate pattern recognition receptors (PRRs) perceive DAMP molecules, which enlarges the lasting efficacy of anticancer drugs by increasing the maturation and recruitment of APC cells (Ahmed and Tait, 2020). The introduction of ICD in cancer immune therapy has predominant clinical significance because the anti-tumor immune responses of ICD exert an important influence on the efficacy of long-term anticancer immunity.

The excessive buildup of nonsense mutations within genes usually generates pseudogenes, which used to be deemed as non-functional because they were short of protein-coding ability or cellular gene expression. However, there is overpowering proof manifesting that pseudogenes are identified as critical hallmarks in tumor initiation, progression, and therapy (Ye et al., 2021; Liu et al., 2022). For instance, pseudogene *WTAPP1* is found to be strongly expressed due to the m^6^A modification and high *WTAPP1* displays a worse prognosis in pancreatic ductal adenocarcinoma (PDAC). *WTAPP1* RNA contributes to the malignant phenotypes of PDAC cells by increasing its protein-coding counterpart (WTAP) translation, which intensifies the Wnt signaling (Deng et al., 2021). Increasing research has developed a reliable pseudogene-related prognosis signature with excellent performance. Tang and Zhuge (2021) generated a prognostic signature based on 9 immune-related pseudogenes and has high efficacy in predicting prognosis and immune response in endometrial cancer (EC).

Herein, we first depicted the role of ICD-related genes in AML and defined two AML clusters according to 33 ICD-related gene expression patterns. The association between classification and tumor immunotherapy was also explored. More importantly, we identified ICD-related pseudogenes and created a prognosis signature based on the critical pseudogenes. Also, we corroborated the prognostic significance of this signature and the potential value of the ICD-related pseudogenes signature in immunotherapy and chemotherapy.

## 2 Materials and methods

### 2.1 Data collection

The transcriptome and clinical data (Supplementary Material S1) of AML were downloaded from the TCGA (https://portal.gdc.cancer.gov/) database, while the GSE71014 dataset was acquired from the GEO website (https://www.ncbi.nlm.nih.gov/geo). GSE71014 is an RNA sequencing dataset containing 104 AML patients. The single-cell RNA sequencing dataset AML_GSE116256 was downloaded from the TISCH website (http://tisch.comp-genomics.org/) and contains 5 healthy control donors and 16 AML patients. TCGA-LAML served as the training dataset and GSE71014 was the validation set. AML_GSE116256 was then used to validate gene expression at the single-cell level. After merging two datasets into a meta dataset, the batch correction was performed with the sva package in the R project (4.1.1). Principal component analysis was used to visualize the data distribution of combining gene expression patterns. At the same time, 33 ICD-related genes were identified according to a previous search from previously published literature for the following research (Garg et al., 2016). All statistical analyses were conducted in the R project.

### 2.2 Cluster analysis and survival analysis

To explore the biological functions exerted by ICD-associated genes in AML patients, we separated the overall AML samples into different subsets by using the ConsensusClusterPlus package in R based on 33 ICD genes expression levels in the merging dataset. The classification had the best performance when the parameter consensus matrix k was 2. As a result, AML patients were divided into two clusters. Then Kaplan-Meier curve was applied to display the survival rates of two clusters. Also, the expression data of 33 ICD genes in two AML clusters were extracted from the merging dataset. Box plots and heatmap were employed to visualize the results.

### 2.3 Immune microenvironment feature analysis

A variety of immune cells make up the major cellular components in the tumor environment (TME) ecosystem and are engaged in maintaining TME hemostasis. In the current study, we obtained the relative amounts of immune cells in two clusters by using GSVA package of single sample gene set enrichment analysis (ssGSEA) algorithm. Stromal components are indispensable members in reshaping TME and assessing stromal infiltration degree provides important guidance for predicting immunotherapy efficacy. ESTIMATE algorithm contributes to the quantification of immune and stromal components in TME. Herein, the immune score and stromal score of two ICD clusters were calculated by ESTIMATE and we examined the score differences in two clutters.

### 2.4 Prediction of immunotherapy and chemotherapy response

A tumor immune dysfunction and exclusion (TIDE) scoring system has been developed to predict the immunotherapy response in multiple cancer patients (Wu et al., 2021). The computational framework for TIDE is constructed according to two types of tumor immune escape: T cell dysfunction in high levels of cytotoxic T lymphocytes (CTL) and T cell infiltration in low levels of CTL. A reduced TIDE score represents activated immune checkpoint blockade (ICB) response. We compared the TIDE score in two clusters and investigated immune checkpoint gene expression in two clusters. Besides immunotherapy, chemotherapy is a predominant treatment approach in AML. The IC50 value of some common chemotherapeutic agents such as Cytarabine, Midostaurin, and Doxorubicin in AML therapy between two clusters was also compared.

### 2.5 Identification of ICD-associated pseudogenes

A total of 11597 ICD-associated pseudogenes were obtained firstly via the GENCODE, Vega, and Pseudogene.org databases. 980 ICD-associated pseudogenes were finally identified by the screening criteria (*r* > 0.3 and *p* < 0.001) and 153 of 980 pseudogenes were established for the subsequent analysis by taking the intersection with the GSE70714 validation set. The relationship between ICD genes and matching pseudogenes was shown in the Sankey diagram.

### 2.6 Construction of prognostic model identified by ICD-related pseudogenes

The prognostic ability of ICD-related pseudogenes were first evaluated by univariate method. Next Kaplan-Meier analysis was used to consistently pick out the crucial prognostic-related pseudogenes. 33 ICD-related pseudogenes had a significant impact on AML survival. Then the gene model with the highest efficacy was ensured using least absolute shrinkage and selection operator (lasso) cox risk model (iteration = 1000). The 15-genes model was found to produce the best performance and we developed the prognostic model.

### 2.7 Efficacy validation of the prognostic model

According to the formula determined by 15 pseudogenes expressions in the AML sample, the risk score of each AML sample was acquired by lasso regression. With the median of all pseudogenes scores, the AML samples could be categorized into low and high ICD pseudogene groups. The KM survival curves were used to demonstrate the survival differences between the two groups in both training and validation datasets. The prediction accuracy of the ICD pseudogene model was displayed by the area under the curve of receiver operator curve (ROC) in two groups. Univariate and multivariate analyses were introduced to evaluate the prognostic potential of this ICD pseudogene model. A nomogram combining clinical features was generated to predict the survival rates of AML patients. The calibration curve analysis was employed to examine the prediction accuracy of the ICD pseudogene model.

### 2.8 Prediction of chemotherapy and immunotherapy efficacy by prognostic model

For chemotherapy, the pRRophetic package can predict phenotype from gene expression data and predict drug sensitivity in external cell lines. The pRRophetic package was used to investigate the correlation of 251 chemotherapeutic drugs with the prognostic model. For the immunotherapy, the differences in immune checkpoint gene expression between high and low-risk groups and TIDE scores were compared between high and low-risk groups.

### 2.9 Statistical analyses

R software, version 4.1.1, was used to conduct statistical analyses. The differences between the two groups were compared using the Wilcoxon test, while the differences between the numerous groups were examined using the Kruskal–Wallis test. Statistical significance was defined as *p* ≉< 0.05 (*p < 0.05, **p < 0.01 and ***p < 0.001).

## 3 Results

### 3.1 Two clusters identified by the ICD genes

**FIGURE 1:**
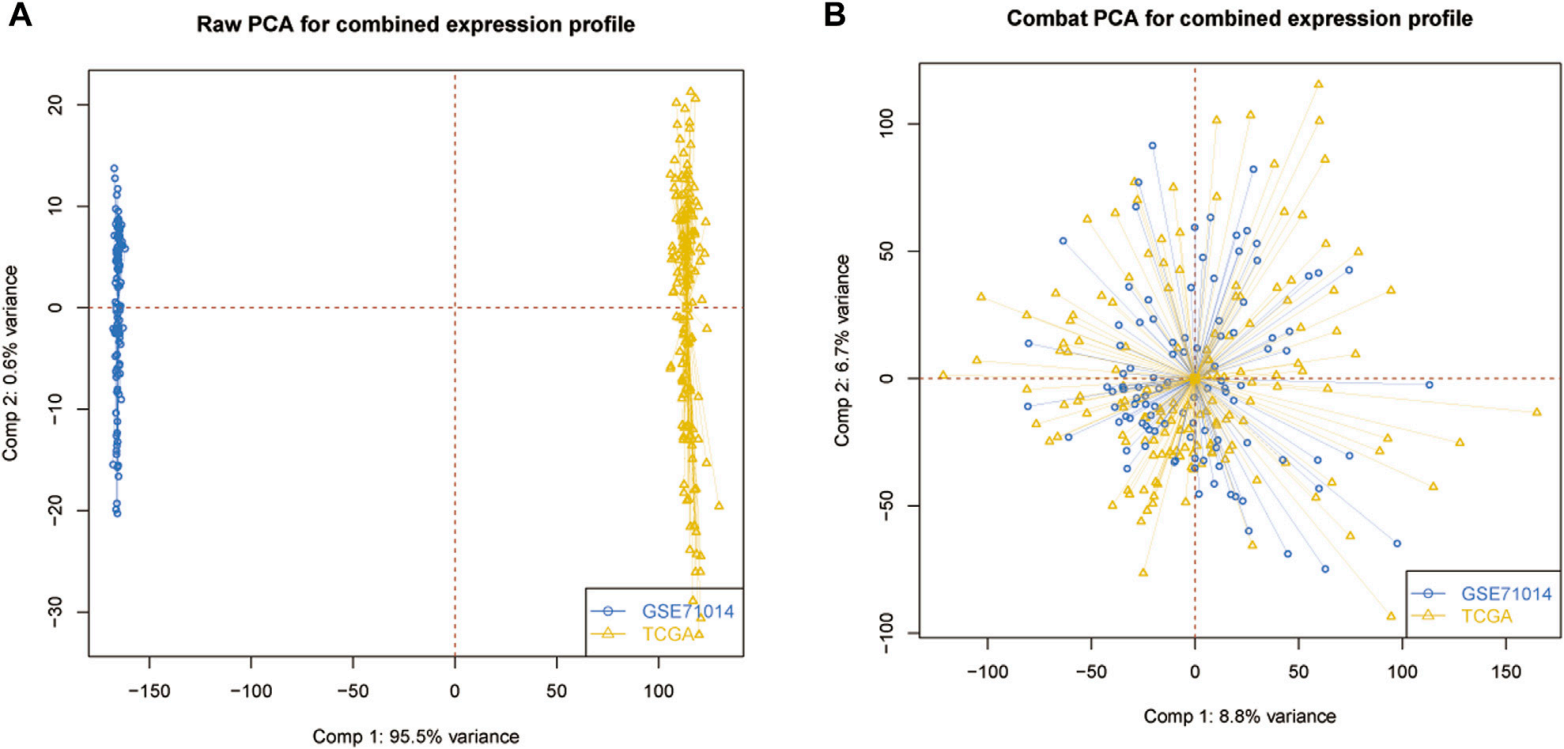
Data processing. **(A)** Original data distribution pattern. **(B)** Mixed data distribution pattern after PCA analysis.

**FIGURE 2:**
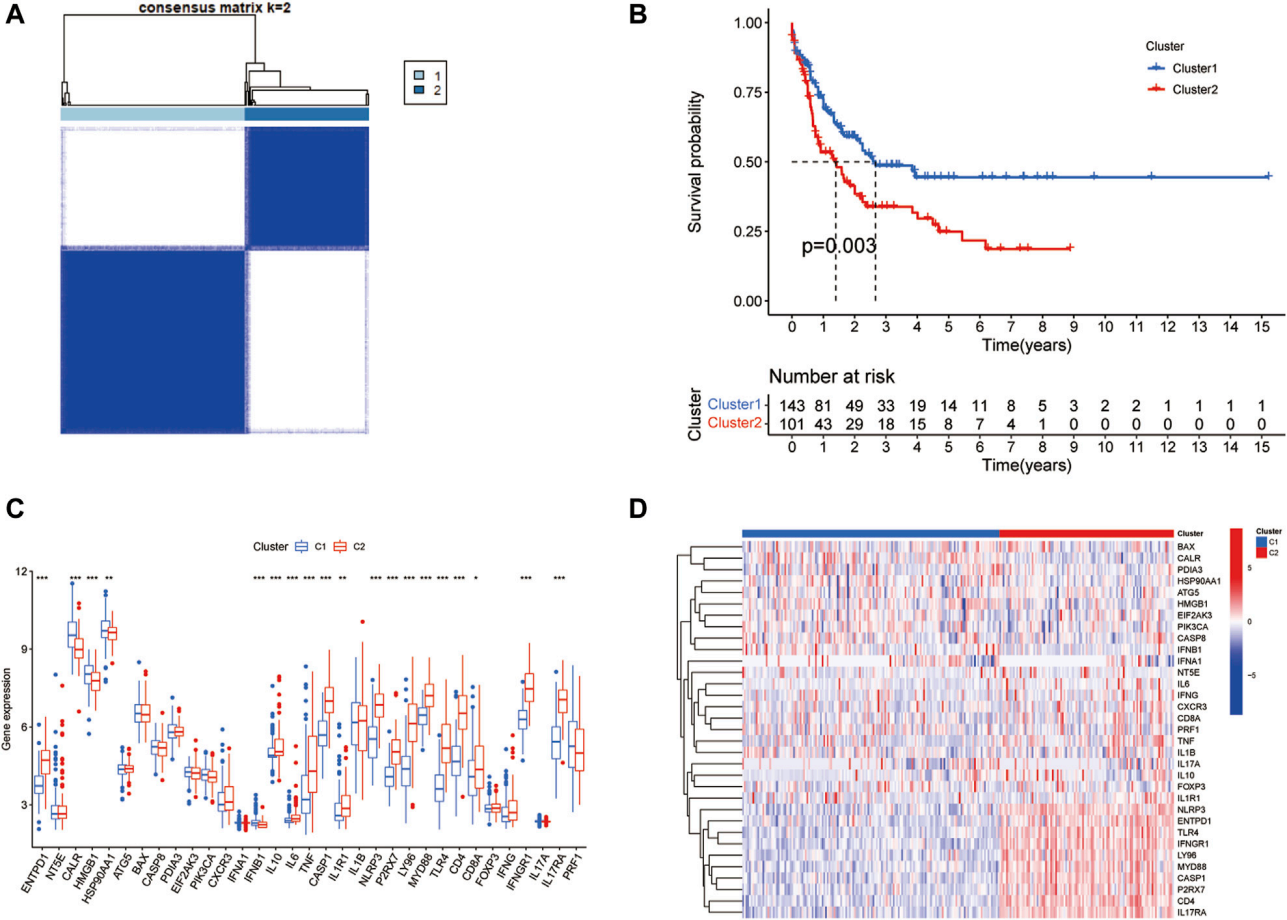
Two clusters identified by ICD genes. **(A)** The plot of consensus clustering of LAML samples with k = 2. **(B)** Survival curve of two clusters. **(C)** The box plot of expression levels of 33 ICD genes in two clusters. **(D)** Expression heatmap of 33 ICD genes in two clusters.

The PCA analysis was introduced to abolish the batch effect of the meta dataset consisting of the GSE71014 dataset and TCGA-LAML cohort (Figures 1A, B). According to recently published research, 33 ICD-related genes were obtained. Then by applying the consensus clustering analysis (Supplementary Figure S1), LAML samples were classified into two distinct clusters (Figure 2A). And KM survival curve showed that the patients in the C1 cluster had a higher survival rate than the C2 cluster (Figure 2B). The expression levels of ICD genes altered excessively across the two clusters (Figures 2C, D). Among them were NLRPJ, ENTPD1, TLR4, FNGR1, LY96, MYD8S, CASP1, P2RX7, CD4, and IL17RA expression levels were significantly higher in the C2 cluster than in the C1 cluster (Figure 2D), implying the critical but complex role of these ICD genes in LAML subtypes.

### 3.2 Identification of tumor microenvironment features in two LAML subgroups

**FIGURE 3:**
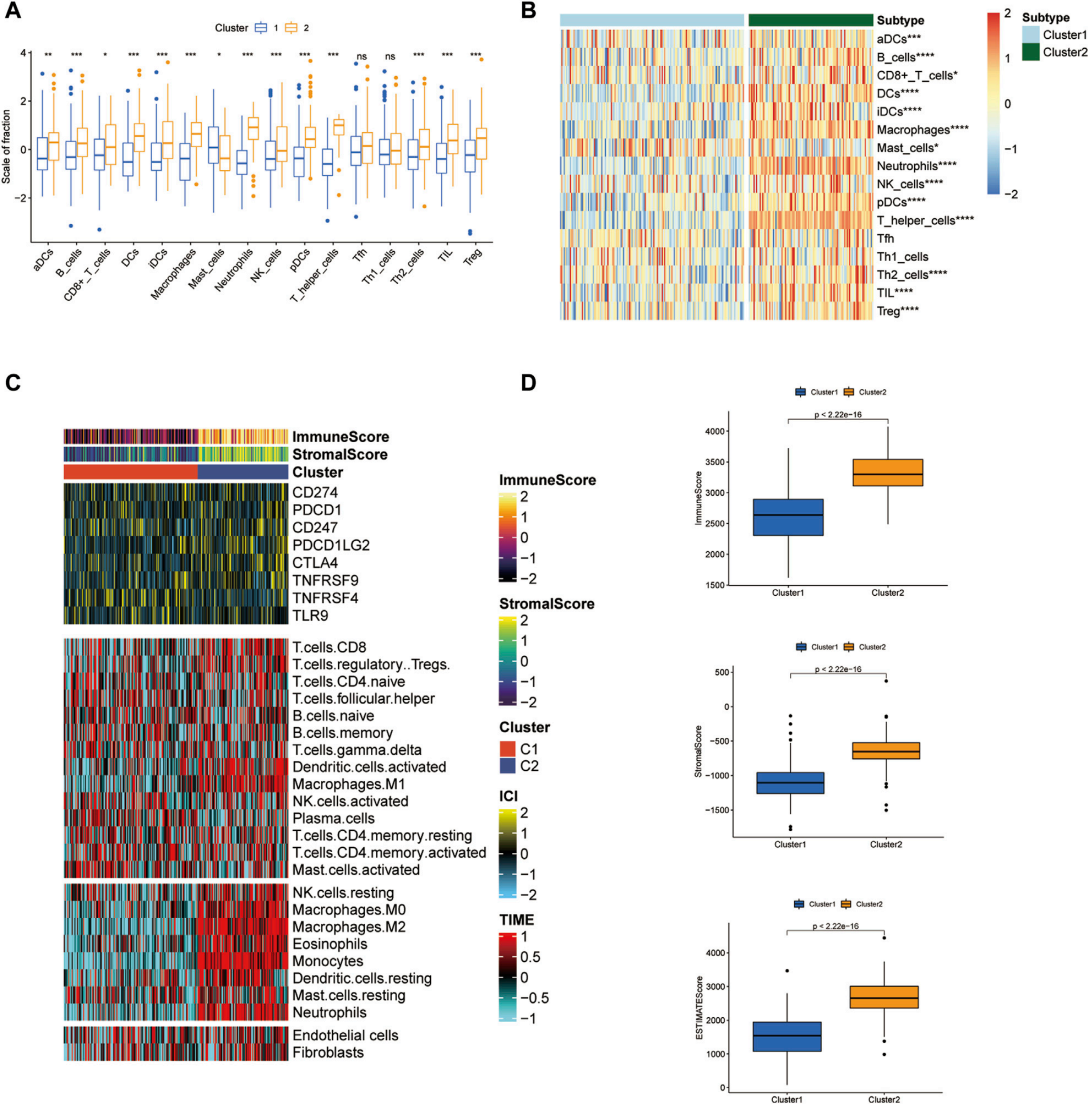
Tumor microenvironment features analysis in two LAML subgroups. **(A)** The box plot of immune cell proportions in two clusters. **(B)** The heatmap of immune cell proportions in two clusters. **(C)** The immune score, matrix score, and immune checkpoint expression in two clusters. **(D)** ESTIMATE score of two clusters.

TME ecosystem consists of various immune cells with cancer-promoting or anti-cancer functions and matrix components, together with tumor cells. The immune cell infiltration degree exerts a decisive impact on the immunotherapeutic outcome such as the immune checkpoint blockade. Given this observation, we surveyed the overall immune cell amounts in two subtypes by using the ssGSEA algorithm. Our findings suggested that the amounts of most immune cells altered significantly across two clusters and were highly enriched in the C2 cluster (Figures 3A, B). For instance, B cells, CD8^+^ T cells, DC cells, macrophages, neutrophils, NK cells, CD4^+^ T cells, and Treg cells were enormously increased in the C2 cluster. Furthermore, LAML-specific immune cell infiltration status was next investigated. We quantified the infiltration level of 24 microenvironmental cell types and investigated the expression of immune checkpoint genes in two LAML subgroups (Figures 3C, D). The immune score, matrix score, and ESTIMATE were higher in the C2 cluster, which was consistent with the ssGSEA result. These research findings confirmed the higher immune infiltration level of the C2 cluster. In addition, the immune checkpoint expression landscape of the two clusters was distinguished from each other.

### 3.3 Immune checkpoint analysis and prediction of chemotherapeutic agents of two clusters

**FIGURE 4:**
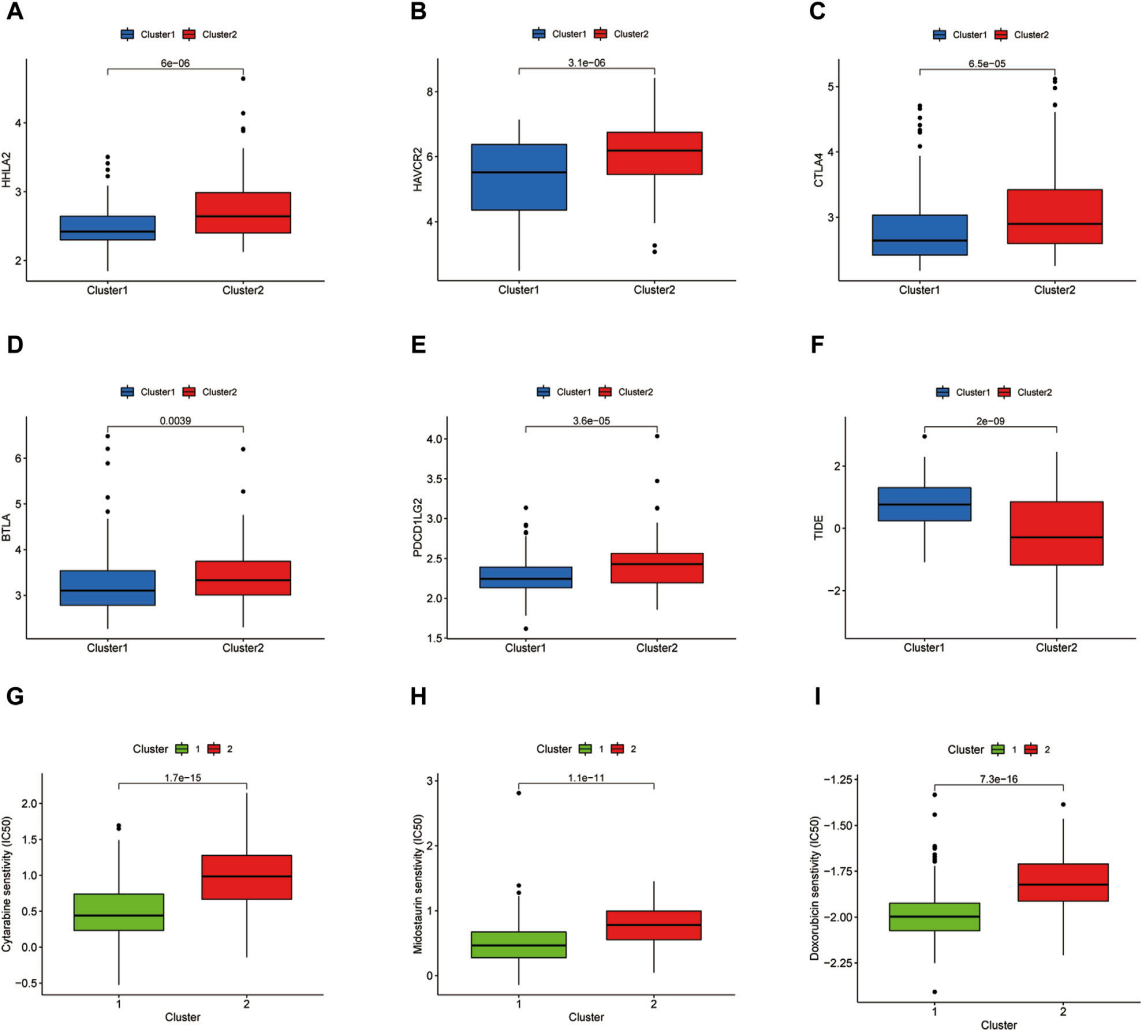
Immune checkpoint analysis and prediction of chemotherapeutic agents. **(A–E)** Several immune checkpoint expression levels in two clusters. **(F)** TIDE score of two clusters. **(G–I)** IC50 values of Cytarabine, Midostaurin, and Doxorubicin in two clusters.

The immune checkpoint expression landscape of the two clusters was distinguished from each other. HHLA2, HAVCR2, CTLA4, BTLA, and *PDCD1LG2* were upregulated in the C2 cluster (Figures 4A–E). The C2 cluster obtained a higher tumor immune dysfunction and exclusion (TIDE) score, which represented an enhancement of immune evasion ability and a worse response to ICB therapy (Figure 4F). As a result, the patients in the C2 cluster could benefit from the ICB therapy and have a better prognostic outcome relative to the patients of the C1 subset. Chemotherapy has emerged as an effective anti-cancer therapeutic strategy in reliving AML progression. Cytarabine, Midostaurin, and Doxorubicin have been considered the conventional chemotherapeutic drugs in anti-AML treatment. The IC50 values of three chemotherapeutic agents for LAML were then predicted and the results showed that the C1 cluster was more sensitive to treatment with three drugs, demonstrating that C1 cluster populations were suitable for the chemotherapy (Figures 4G–I).

### 3.4 Establishment of the prognostic model identified by ICD-associated pseudogenes

**FIGURE 5:**
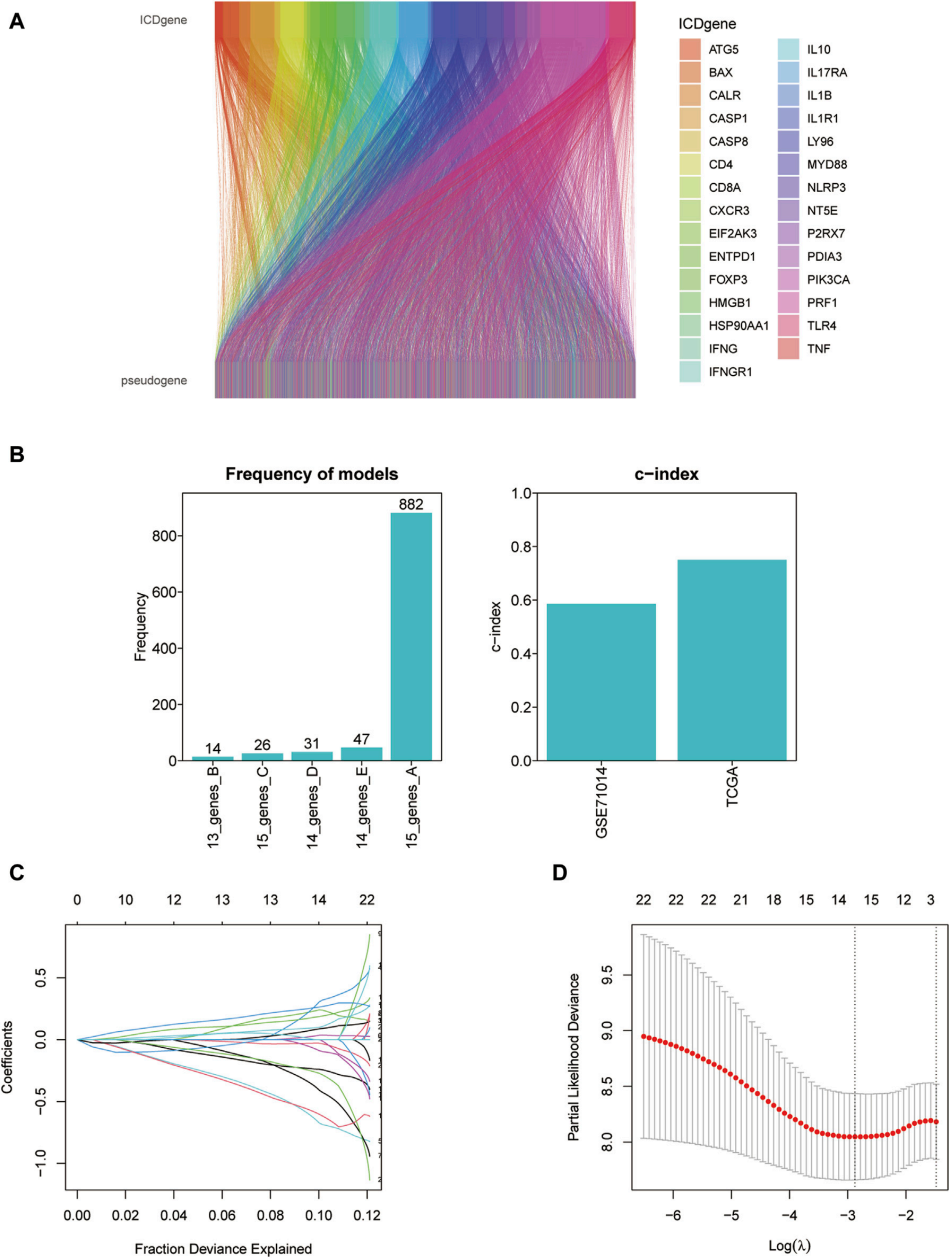
Constructing the prognostic model based on ICD-associated pseudogenes. **(A)** The Sankey diagram of the relationship between ICD genes and ICD-associated pseudogenes. **(B)** The frequency differences of models containing different gene numbers. **(C)** Path diagram of lasso coefficients for 15 ICD-related pseudogenes. **(D)** Cross-validation curves of the lasso.

We screened ICD-associated pseudogenes by performing Pearson correlation analysis (*r* > 0.3 and *p* < 0.001) and 11597 pseudogenes were identified. Then by taking the intersection with the genes of the GSE71014 dataset, 153 pseudogenes were obtained in the subsequent analysis. The relationship between ICD genes and ICD-associated pseudogenes was shown in the Sankey diagram (Figure 5A). The multivariate cox regression analysis and Kaplan-Meier analysis were introduced to find out the prognosis-related ICD pseudogenes, respectively. 23 overlapping pseudogenes between multivariate cox regression and survival analysis were identified for the following investigation. To increase the predictive value of ICD pseudogenes in determining the clinical outcomes of LAML, we developed a risk model by implementing the lasso cox regression method to the 23 ICD pseudogenes. These 23 ICD-related pseudogenes were then subjected to cox proportional risk regression with 10-fold cross-validation to generate the best genetic model numbers. We performed a total of 1000 iterations, including 5 genomes, for further analysis (Figure 5B). The gene model with 15 ICD-associated pseudogenes had the highest frequency (882) compared to the other 4 gene models. Therefore, this 15 genes model was most suitable for constructing a prognostic signature (Figures 5C, D). In AML_GSE116256, cells are distinguished into a total of 13 subtypes, which are HSC cell, Malignant cell, Monocyte cell, Progenitor cell, GMP cell, CD4Tn cell, EryPro cell, Promonocyte cell, CD8Tcm cell, B cell, Tprolif cell, NK cell, Plasma cell (Supplementary Figure S2A). At the single-cell level we verified the expression of a total of 8 genes, namely, FAHD2CP, NAPSB, ADCY10P1, WHAMMP3, SUZ12P1, SDHAP3, RPS10P7, PDCD6IPP2 (Supplementary Figures S2B–I). Among them, NAPSB was significantly highly expressed in Malignant cells, suggesting that NAPSB may be a marker gene for Malignant cells.

### 3.5 Predictive efficacy validation of the prognostic model

**FIGURE 6:**
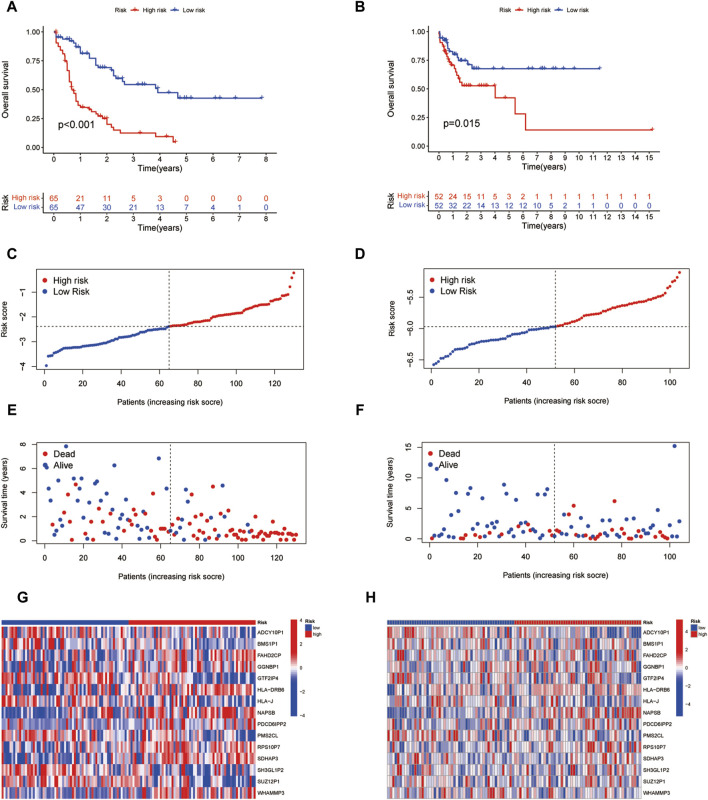
Efficacy validation of the prognostic model. **(A,B)** Survival analysis of two risk subgroups in the TCGA-LAML cohort and GSE71014 cohort. **(C,D)** Distribution of risk scores in each sample between two risk subgroups in TCGA-LAML cohort and GSE71014 cohort. **(E,F)** Distribution of survival status and survival time of each sample between two risk subgroups in the TCGA-LAML cohort and GSE71014 cohort. **(G,H)** Gene expression of 15 ICD pseudogenes between two risk subgroups.

According to the coefficients of 15 ICD-associated pseudogenes, we calculated the ICD related risk score of each LAML sample relying on the prognostic model. Based on the median ICD risk score, LAML samples were divided into low or high ICD groups. Kaplan-Meier survival analysis showed that patients in the high ICD pseudogenes group had worse overall survival rates than those in the low ICD pseudogene group in both the training (TCGA-LAML cohort) and test datasets (GSE71014 cohort), demonstrating that the low ICD-associated pseudogenes group had a better prognosis outcome (Figures 6A, B). More death events were observed in the high ICD-associated pseudogenes group, suggesting that the increasing ICD pseudogenes level reflected the unfavorable prognosis of patients with LAML (Figures 6C–H).

### 3.6 The prognosis signature identified by 15 ICD pseudogenes was an independent prognostic factor in LAML

**FIGURE 7:**
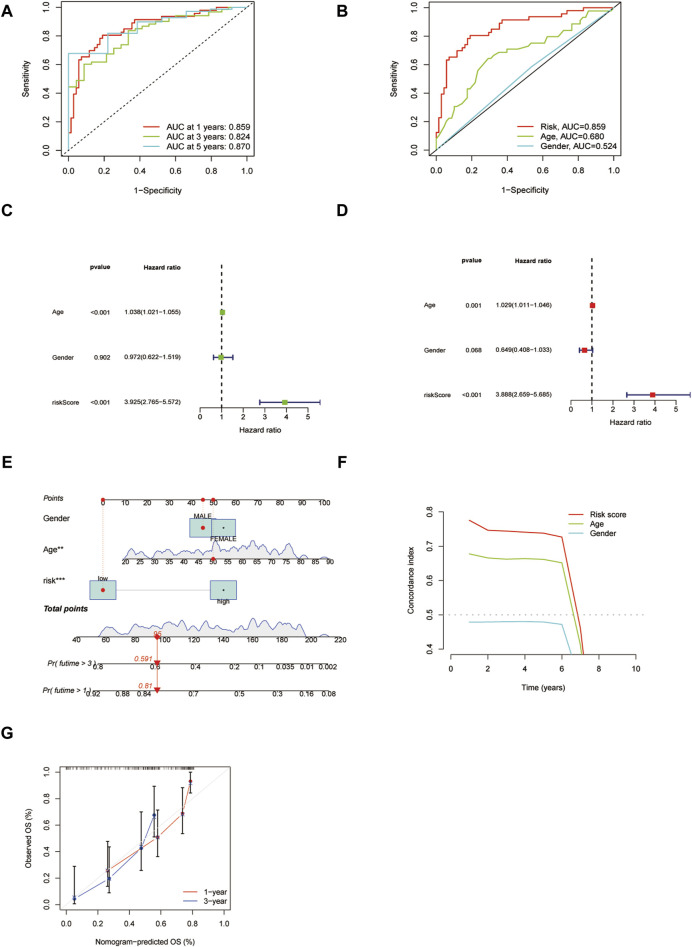
The validation of the independent prognostic potential of the risk model. **(A)** ROC analysis of the prognostic model at predicting 1, 3, and 5 years. **(B)** ROC analysis of the risk model and clinical features. **(C,D)** Univariate and multivariate cox regression analyses of the risk model, respectively. **(E)** Clinical nomogram of the risk model. **(F)** The C-index plot of the nomogram. **(G)** The calibration curve for predicting survival at 1 year and 3 years.

To assess the predictive efficacy of 15 ICD-associated pseudogenes model, we examined the independent prognostic potential of ICD prognostic model by conducting the ROC analysis. The ICD pseudogene model had higher efficacy (AUC values were >0.8) in the training group (Figure 7A). The AUC value of the 15 pseudogene model was higher than that of general AML clinical characteristics such as age and gender (Figure 7B), indicating that the prognostic model carried good predictive value for the prognosis of LAML patients. We then performed univariate and multivariate cox regression analyses to assess the independent predictive potential of the prognostic model. The results showed that our risk score could be used as an independent prognostic factor (Figures 7C, D). By combining the independent prognostic factor, we constructed a nomogram as a clinically relevant quantitative method by which the mortality in LAML patients could be predicted (Figure 7E). By summing the scores of each prognostic parameter, a total score value would be obtained for each patient. A higher final score corresponds to a worse prognosis outcome. The time-dependent C-index curve showed a high predictive power of the columnar plot (Figure 7F). The calibration curve showed that the column model has an ideal diagnostic efficacy (Figure 7G).

### 3.7 Predictions of chemotherapeutic agents and ICB analysis in two risk groups

**FIGURE 8:**
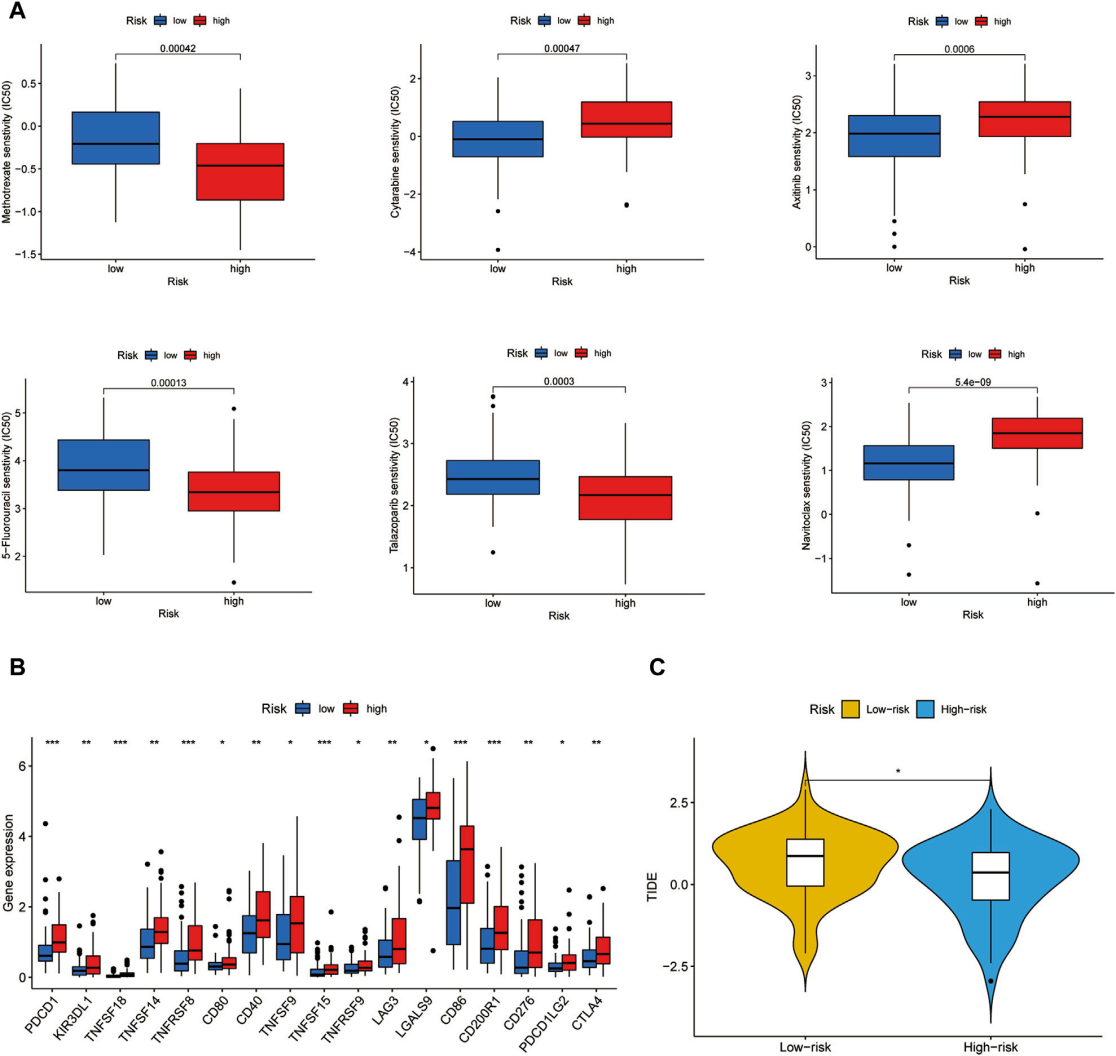
Chemotherapeutic agents sensitivity and ICB analysis. **(A)** IC50 values of 6 chemotherapeutic drugs in two risk groups. **(B)** Immune checkpoints expression in two risk groups. **(C)** TIDE score of two risk groups.

To depict the underlying association between chemotherapy and the risk model, we investigated the sensitivity of 6 conventional chemotherapeutic agents in two clusters by calculating the IC50 values of each sample. By comparing the differences in IC50 values between the two risk groups, it was found that Cytarabine and Axitinib were sensitive in the high-risk group while Methotrexate, 5-Fluorouracil, Talazoparib, and Navitoclax were sensitive in the low-risk group (Figure 8A). Immune checkpoint analysis showed that most immune checkpoints genes such as *PDCD1* and *CTLA4* were significantly increased in the high-risk group (Figure 8B). In agreement with this point, the high-risk group acquired a lower TIDE score than the low-risk subtype, confirmed by the TIDE analysis results (Figure 8C). As a result, ICB therapy would produce high clinical efficacy and lead to a good prognosis outcome in high-risk groups.

## 4 Discussion

Regulated cell death (RCD) occurs in mammalian cells when the microenvironmental distribution signals surpass the maximal processing potential of cellular homeostasis, which promotes cytoprotecting molecular mode changing into cytotoxic cascades (Galluzzi et al., 2018; Bedoui et al., 2020). As the specific variant of RCD, ICD is triggered by the stress signals such as epigenetic modifiers, tumor cell lysis molecules, radiation therapy, traditional chemotherapeutic agents even specific anticancer agents (tyrosine kinase inhibitors, cetuximab) (Galluzzi et al., 2017; Fucikova et al., 2020; Galluzzi et al., 2020). Recent research has reported that the successful benefits of anti-cancer drugs are derived from the increasing tumor-targeting immune response caused by ICD (Petroni et al., 2021). Combining immunogenic therapy with immunotherapy improves survival time and achieves better clinical outcomes in human cancers (Boumelha et al., 2022). Wang et al. (2021) have defined two head and neck squamous cell carcinoma (HNSSC) subtypes based on several ICD-related genes and find that the high-ICD subtype obtains a better prognosis and benefits from the immune therapy. Chen et al. (2024), Zhong et al. (2024), Sheng et al. (2023) have explored the role of ICDs in AML by distinguishing AML subtypes and establishing prognostic models, but establishing a link with pseudogenes has not been investigated. It is advantageous to develop a reliable immunogenic biomarker that holds great promise for treating malignancies. Here we pinpointed the prognosis and immunotherapy implication of ICD in LAML.

In the current study, we emerged the TCGA-LAML and GSE71014 cohort and classified LAML into two clusters (C1 and C2) premised on 33 ICD-associated gene expression levels by consensus clustering analysis. The C2 cluster had higher ICD gene expression and immune cell infiltration, implying the C2 cluster benefits immune therapy. Notably, NK cells and CD8^+^ T cells were more enriched in the C2 cluster. NK cells exert a predominant tumor-suppressing function by indirectly generating multiple cytotoxic cytokines and directly killing tumor cancer. Recent clinical trial data reveal that cytokine-induced memory-like NK cells derived from the same-donor hematopoietic cell transplantation (HCT) achieved a complete response and persisted for over 2 months in relapsed/refractory AML patients. ML NK cells devastate tumor cells by producing granzyme B and perforin and expressing *CD16* and *CD57* (Berrien-Elliott et al., 2022). Vitro and *in vivo* evidence suggested that the novel monoclonal antibody (T-1A5) targeting immune checkpoint B7-H3 plays an immune-suppressive role by endorsing the cytotoxicity activity of NK cells. Combining T-1A5 with NK cells significantly improves survival time in AML (Tyagi et al., 2022). CD8^+^ T cell exhaustion is present in the bone marrow in the murine receiving the allogeneic bone marrow transplantation. Inhibiting the CD8^+^ T cells with exhaustion phenotype before allogeneic bone marrow transplantation spares T cells from obtaining a stem-like memory phenotype, which magnifies the antitumor immunity response by swelling cytokine signaling gene expression such as IL18 receptor via increasing the chromatin accessibility (Minnie et al., 2022). Furthermore, we noted that immune checkpoint *HAVCR2* (also termed TIM3) is highly expressed in the C2 cluster. In line with our research results, Rakova et al. (2021) have pointed out that TIM-3 is strongly expressed in the NK cells in AML patients and encourages NK cell cytotoxicity and superior effector functions, which improves survival outcomes in AML patients. These findings demonstrated that the C2 cluster could achieve high efficacy in immune therapy relative to the C1 cluster, also confirmed by the TIDE analysis results. Of course, the C1 cluster subgroup would benefit from traditional chemotherapeutic drugs including Cytarabine, Midostaurin, and Doxorubicin.

Considering the significant role of pseudogenes in determining cancer progression and clinical outcome, we surveyed the overall function landscape of ICD-related pseudogenes in AML. 153 pseudogenes were identified to be associated with 33 ICD genes and 23/153 pseudogenes were found to be related to AML prognosis by univariate regression and Kaplan-Meier analysis. Hinging on lasso regression analysis, we found that 15 pseudogenes were suitable for generating a prognostic model with high efficacy. The AML sample was categorized into high and low-risk groups according to the risk score calculated by the prognostic model. Survival analysis in both TCGA-LAML and GSE71014 cohorts illuminated that the high-risk group had a reduced overall survival rate and more death events. Increased risk score reflected the unfavorable prognosis for AML. These results verified the high efficacy of our prognostic model in evaluating the prognosis of AML patients.

To probe whether 15 ICD-related pseudogenes serve as an independent prognostic index, the prognostic influence of 10 pseudogenes was exemplified. ROC analysis certified that our prognostic model did the best performance in predicting prognosis relative to clinical features such as age and gender. Cox analysis supported that 15 ICD-related pseudogenes could be considered as an independent prognostic factor in AML. To better dissect the diagnostic value of 15 ICD-related pseudogenes, we created a clinical nomogram based on 15 pseudogenes. Results hinted that the risk score calculated by the prognostic model could play a determining role in survival assessment combined with clinical features. The c-index and calibration curve confirmed that the clinical nomogram had higher predictive ability and sensitivity.

Finally, we compared the IC50 values of 6 conventional chemotherapeutic agents in two risk subgroups. Cytarabine and Axitinib were found to be perceptive to the high-risk group while Methotrexate, 5-Fluorouracil, Talazoparib, and Navitoclax were hypersensitive to the low-risk group. Smarmily, immune checkpoints such as *PDCD1, TNFSF18, TNFRSF14, CD40, LAG3, LGALS9, CD86, CD200R1, CD276,* and *CTLA4* were strongly expressed in the high-risk group. The high-risk group was hyper-sensitive to the immune checkpoint inhibitor therapy. Moreover, in line with this point, the TIDE score of the high-risk group was reduced, hinting at the increased anti-tumor immune response to the immunotherapy.

## 5 Conclusion

Our research successfully categorized the TCGA-AML and GSE71014 samples into two subtypes premised on 33 ICD-related genes and explicated the tumor immune environment and therapy differences between the two clusters. Furthermore, we created a stable prognostic model using 15 ICD-related pseudogenes and this prognostic signature performed excellently in determining the clinical efficacy of immunotherapy and chemotherapy. Although clinical validation of our findings is still needed, for the time being, our study points out the new significance of ICD in the prognosis and precision treatment of acute myeloid leukaemia, and provides new ideas for the treatment of AML.

## Data Availability

The original contributions presented in the study are included in the article/Supplementary Material, further inquiries can be directed to the corresponding author.

## Supplementary material

SUPPLEMENTARY FIGURE S1 Consensus clustering results **(A)** Cumulative Distribution Function (CDF) Plot, **(B)** Delta Area Plot, **(C)** Tracking Plot.

SUPPLEMENTARY FIGURE S2 Gene expression at the single cell level **(A)** UMAP plot of different cells **(B–I)** Expression of FAHD2CP, NAPSB, ADCY10P1, WHAMMP3, SUZ12P1, SDHAP3, RPS10P7, PDCD6IPP2 at the single cell level.

SUPPLEMENTARY MATERIAL S1 The clinical variables for AML patients.
