# Supplementary figures and images for: Immunogenic cell death (ICD) genes predict immunotherapy response and therapeutic targets in acute myeloid leukemia (AML)
Source: Front Genet. 2024 Aug 14;15:1419819. doi: 10.3389/fgene.2024.1419819 (PMC11349646; doi:10.3389/fgene.2024.1419819)

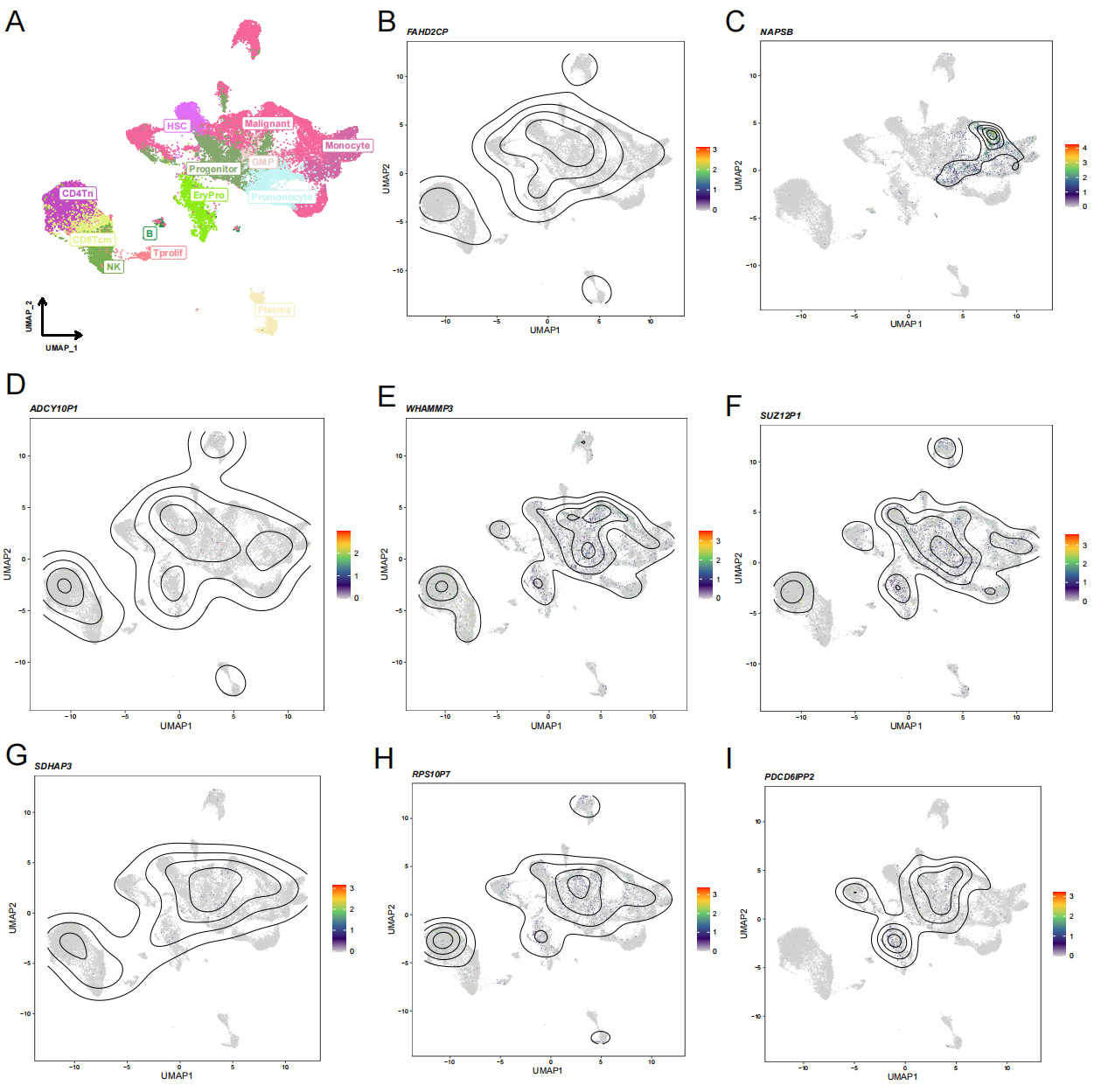

Supplement: Supplementary file 2 [file Image2.TIF]

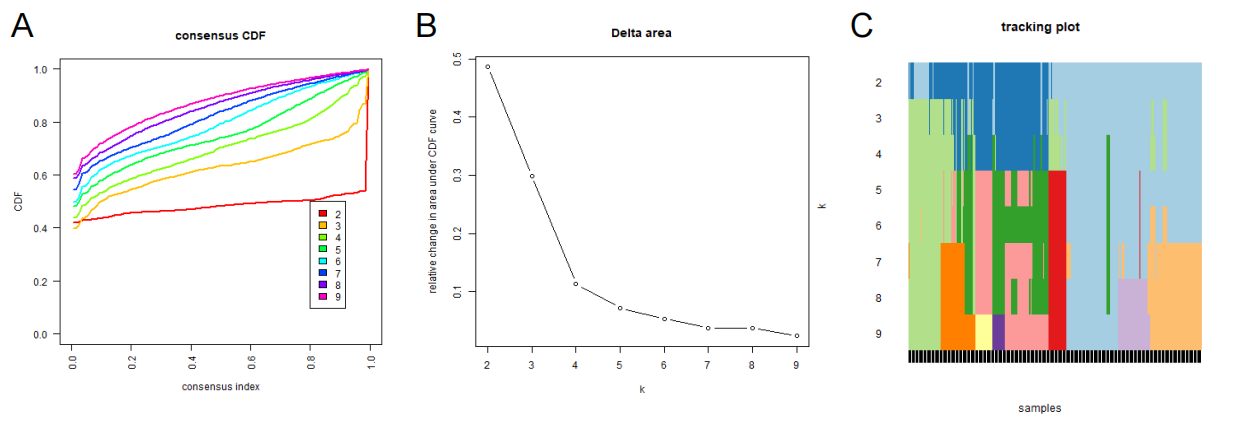

Supplement: Supplementary file 3 [file Image1.TIF]
